# A collection of the novel coronavirus (COVID-19) detection assays, issues, and challenges

**DOI:** 10.1016/j.heliyon.2021.e07247

**Published:** 2021-06-06

**Authors:** Erfan Rezvani Ghomi, Fatemeh Khosravi, Ali Mohseni-M, Nooshin Nourbakhsh, Mahsa Haji Mohammad Hoseini, Sunpreet Singh, Mikael S. Hedenqvist, Seeram Ramakrishna

**Affiliations:** a Center for Nanotechnology and Sustainability, Department of Mechanical Engineering, National University of Singapore, Singapore, 117581, Singapore; b Executive Vice President and Chief Food Safety Officer, American Foods Group, LLC, 500 South Washington St., Green Bay, WI, 54301, USA; c Dir. Ag. Group. Qoqnoos – Phoenix Project Incorporated, USA; d Yong Loo Lin School of Medicine, Department of Medicine, National University of Singapore, Singapore, 119228, Singapore; e Emergency Department, Paramedical Faculty, Qom University of Medical Sciences, Qom, 3713649373, Iran; f Department of Fibre and Polymer Technology, School of Engineering Sciences in Chemistry, Biotechnology and Health, KTH Royal Institute of Technology, Stockholm, 100 44, Sweden

**Keywords:** COVID-19, SARS-CoV-2, Pandemic, Rapid detection techniques, Polymerase chain reaction, Safety, Technology

## Abstract

The global pandemic of COVID-19 has rapidly increased the number of infected cases as well as asymptomatic individuals in many, if not all the societies around the world. This issue increases the demand for accurate and rapid detection of SARS-CoV-2. While accurate and rapid detection is critical for diagnosing SARS-CoV-2, the appropriate course of treatment must be chosen to help patients and prevent its further spread. Testing platform accuracy with high sensitivity and specificity for SARS-CoV-2 is equally important for clinical, regional, and global arenas to mitigate secondary transmission rounds. The objective of this article is to compare the current detection technology and introduce the most accurate and rapid ones that are suitable for pandemic circumstances. Hence, the importance of rapid detection in societies is discussed initially. Following this, the current technology for rapid detection of SARS-CoV-2 is explained and classified into three different categories: nucleic acid-based, protein-based, and point of care (PoC) detection testing. Then, the current issues for diagnostic procedures in laboratories are discussed. Finally, the role of new technologies in countering COVID-19 is also introduced to assist researchers in the development of accurate and timely detection of coronaviruses. As coronavirus continues to affect human lives in a detrimental manner, the development of rapid and accurate virus detection methods could promote COVID-19 diagnosis accessible to both individuals and the mass population at patient care. In this regard, rRT-PCR and multiplex RT-PCR detection techniques hold promise.

## 1 Introduction

The coronaviruses are a large family of highly diverse single-stranded RNA viruses that cause diseases in humans and animals [1, 2, 3]. Using their so-called spike glycoproteins, these viruses enter the host cells leading to their replication that hinders the host body from its normal functions [4,5]. The first cases of this disease were reported in Wuhan, China, and it was subsequently recognized as a pandemic [6,7]. The disease has currently spread in at least 221 nations, where more than 130.3 million cases are detected with ca. 2.8 million fatalities [8]. The COVID-19 patients infected by SARS-CoV-2 show common flu symptoms such as tiredness, fever, dry cough, running nose, sore throat, and body pain [9,10]. However, some infected cases did not show any symptoms. Although approximately 80% of infected people will recover with no special treatment, it is pernicious for older patients, especially those with other serious diseases such as respiratory illnesses [11].

It was claimed that a SARS-CoV-2 infected individual could potentially transmit the virus to three other persons [12]. Comprehending the detrimental nature of the disease, many governments conducted lockdowns to control the spread of the coronavirus [13,14]. In addition, body temperature screening was carried out in public places to detect infected individuals, albeit the lower effectiveness of this detection method. Considering the highly contagious nature of the virus, it is a top priority of governments to control virus transmission in an expedited manner. Rapid and accurate detection of the infected people may curb the further transmission of the SARS-CoV-2. From the onset of the pandemic, various diagnostic techniques have been utilized for the detection of infected individuals. In this review article, numerous diagnostic approaches for the detection of SARS-CoV-2 are summarized. This article summarized current findings and assays related to detection techniques of the virus, which may be useful to researchers in fighting COVID-19. A web search indicates that a review article has been published regarding diagnosis techniques [12], however, in the current article, the diagnostic issues are explained along with the emphasis on the suggestion of alternatives to them.

## 2 Data collection method

The PubMed, Web of Science (WOS), Science Direct, and Medline databases were used for searching the articles related to COVID-19 and its detection assays. The keywords selected from Medical Subject Headings (MeSH) were COVID-19, SARS-CoV-2, Pandemic, Polymerase Chain Reaction, Safety, and Technology. Only the articles written in English were considered. We then scanned the title and abstract of similar articles, selected the most relevant article, and read them accurately.

## 3 Biological characteristics of SARS-CoV-2

**Figure 1:**
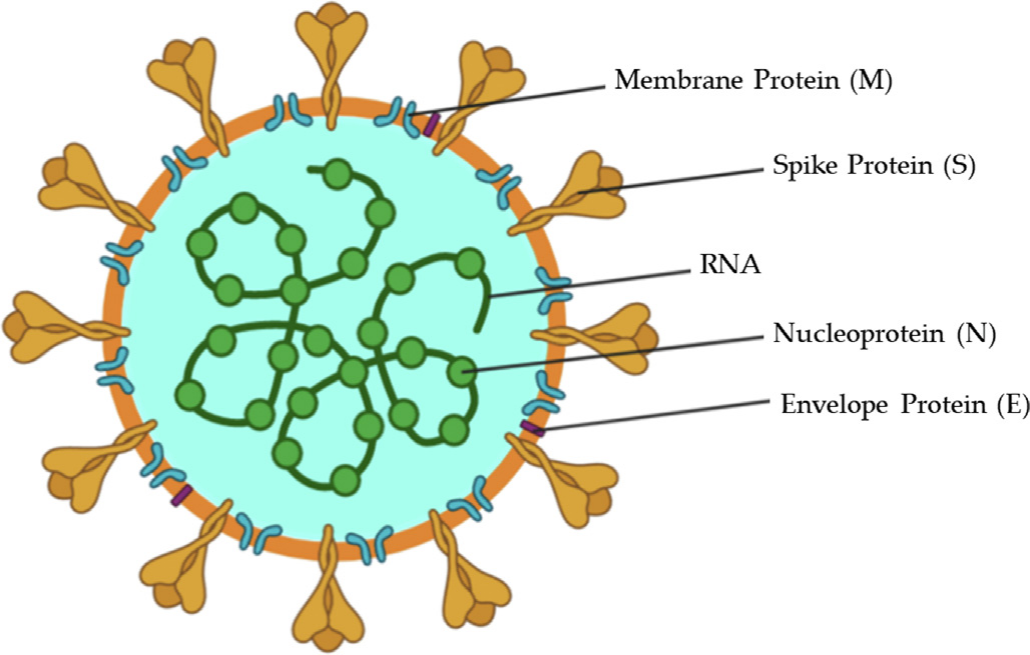
SARS-CoV-2 morphology.

SARS-CoV-2 structural proteins are mainly classified into four major components, including envelope protein (E), membrane protein (M), nucleocapsid protein (N), and spike protein (S), as shown in Figure 1 [12]. The gene S encodes the receptor-binding spike protein that spearheads the main infection mechanism. The gene S of SARS-CoV-2 showed diversity in comparison with all the previously described SARS related-CoVs with less than 75% similarity of the nucleotide sequence [15]. Moreover, the obtained electron micrographs of negatively stained damaged SARS-CoV-2 virus by Zhu et al. (2020) confirmed the virus diameter varied from 60 to 140 nm.

The biology of the virus can assist researchers in perceiving the basic information related to diagnostic techniques for COVID-19 detection. Understanding the virus and its relevant genetic sequence information is required for synthesizing the primers in PCR-based techniques [16]. The spike protein of coronavirus is an important antigen site to detect infection of COVID-19 in serological assays, which are based on identifying the serum antibodies against the S-protein of the virus [17]. In addition, N protein has been found in most case studies infected by SARS-CoV-2 [17].

## 4 Importance of rapid detection

The COVID-19 symptoms, such as respiratory difficulties, fever, dry cough, and so on, are not specifically related to the coronavirus and are common with other diseases [18]. In addition, the COVID-19 infection is highly contagious, and hence, diagnostic tests are quickly needed to verify the virus to warrant subsequent actions [19]. Regarding the urgency of the situation, point of care (PoC) devices are vital and highly important for the rapid detection of SARS-CoV-2 and prevention of its expansion. PoC devices should be rapid, cost-effective, and able to be utilized anywhere [20]. More importantly, they do not require any professional technician [21]. Early detection has an impressive effect on curbing infection [21]. If the detection reporting and lab confirmation occur earlier, the peak number of infected cases will decrease after the first confirmed case. Therefore, it is essential to promote rapid detection techniques to shorten the lab confirmation and response time to stop the spread of the coronavirus [21]. At present, the most common technique for SARS-CoV-2 detection is the real-time reverse transcription-polymerase chain reaction (rRT-PCR) [22]. However, the rRT-PCR technique requires well-equipped laboratories [23]. Moreover, it needs the transportation of the samples to the laboratory.

In emergencies, such as the COVID-19 outbreak, this process is considered as time-consuming, which inadvertently provides an opportunity for the contagious virus to dissipate rapidly. Furthermore, PCR-based techniques show several disadvantages, such as needing expert technicians and being cost-intensive [24].

## 5 Current diagnostic tests for COVID-19

The laboratory methodologies are classified into three different categories: nucleic acid-based detection, protein-based detection, and PoC detection methodology. Although RT-PCR is considered as a leading assay for detecting viral RNA, other diagnostic tools are based on molecular and serological assays and related techniques such as isothermal amplification assays, hybridization microarray assays, amplicon-based metagenomics sequencing, CRISPR technologies, serological and immunological assays.

### 5.1 Nucleic acid-based detection testing

#### 5.1.1 Reverse transcription polymerase-chain reaction (RT-PCR)

**Figure 2:**
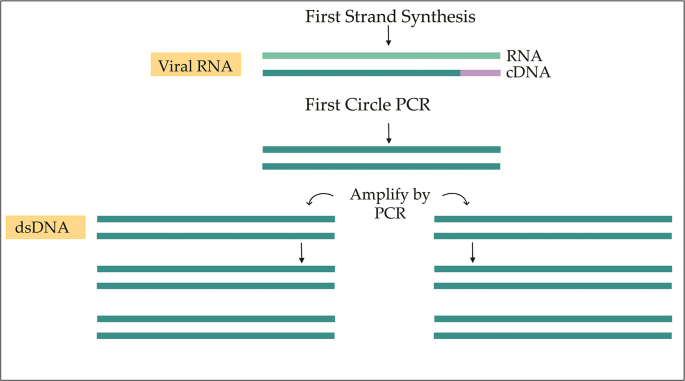
An Overview of Reverse Transcription PCR (RT-PCR): The RT-PCR generates a cDNA copy of the virus RNA.

RT-PCR is a nucleic acid-based detection technique of SARS-CoV-2, which includes two main steps [25]. The first step is the reverse transcription of the single-strand complementary DNA (cDNA) of the SARS-CoV-2 RNA, and the second step contains the amplification of the complementary DNA [25] (Figure 2). RT-PCR is used as a gold standard technique to identify COVID-19, which can amplify small amounts of viral genetic material in a sample [16]. The assay conditions such as temperature reaction, incubation time, and amplification should be optimized, and primer design is critical for the accuracy of the system to identify the unique target sequence [26].

RT-PCR can be performed in two different procedures: one-step and two-step assays. The one-step assay needs optimization of the reverse transcription while the target DNA amplification takes place in one tube, which contains the essential primers for running the RT-PCR reaction. The two-step assay uses at least one tube to run RT-PCR and amplification reactions [16]. Although this assay is more sensitive, flexible, and able to stock cDNA to quantify the multiple targets in comparison with the one-step assay, it is a time-consuming process with a special requirement for the optimization of extra factors and uses more raw material [15,27].

Generally, the one-step assay is preferable over the two-step assay due to fewer human errors such as pipetting, more rapid setup, less time duration, and less contamination between RT and PCR steps [16]. There are several issues associated with using the RT-PCR technique. First, the PCR cannot detect the infection of the recovered asymptomatic patient [15]. The rapid RT-PCR technique is expensive and requires equipped laboratories, expensive instruments, and professional personnel, and the final diagnosis could be released after days. The aforementioned disadvantages and a need for complicated thermal cycle equipment led to the improvement of other rapid diagnostic tests such as an isothermal nucleic acid-based technique for SARS-CoV-2 identification. This technique is a rapid and efficient accumulation way of nucleic acid at a specific constant temperature and is used for various bio-sensing targets such as nucleic acids, proteins, peptides, and ions. The association of the isothermal nucleic acid amplification and the microsystem on any portable device increases its utility in the on-site nucleic acid-based diagnostic assays and also delivers high sensitivity [28]. The other molecular PCR-based technique to identify the widespread COVID-19 is the rRT-PCR, which includes several steps such as the accumulation of patient samples, packing and conveying the samples to the equipped laboratories, and reporting the final results [21]. Similar to the SARS-CoV identification, this technique is currently the most recommended for SARS-CoV-2 [29], and antibody detection techniques are offered as supplemental techniques. An antibody test can measure the concentration of IgG and IgM levels in the blood, serum, and plasma samples to determine if the body is fighting with a pathogen. The most common antibody tests are based on lateral flow type assays (LFA) and enzyme-linked immunosorbent type assays (ELISA) [30]. Antibody responses to infection take days to weeks to be reliably detectable [31]. One of the most important merits of using rRT-PCR is limiting the false rate results attributed to the amplification of product contamination by conducting both amplification and analysis at the same time [32]. rRT-PCR requires a relatively long time to complete the diagnosis, which increases the virus mortality caused by the virus and the risk of more incidence of the infection [21].

#### 5.1.2 Loop-mediated isothermal amplification (LAMP)

Loop-mediated isothermal amplification (LAMP) is a single-tube technique to amplify DNA. It can be considered as an economical alternative technique for disease detection. Reverse Transcription Loop-mediated Isothermal Amplification (RT-LAMP) couples LAMP with a reverse transcription process for detecting RNA. LAMP was recognized as a simple and economical diagnosis approach without the requirement for well-equipped laboratories. LAMP is a nucleic acid-based technique that uses isothermal amplification, obviating the requirement for complicated thermal cycling instruments [33]. In this technique, a DNA polymerase with strand displacement feature will be employed to duplicate the target DNA at 65 °C [34]. In addition, LAMP utilizes four to six primers to detect six to eight regions on the target DNA [33]. LAMP is sensitive and became a reliable isothermal amplification method among biologists to detect pathogens. The LAMP testing technique starts with the patients’ samples in a tube followed by the detection through turbidity [35]. Detection of the amplified DNA is facilitated by adding the pH-sensitive color or fluorescent dye to bind with double-stranded DNA [15]. The important characteristic of the LAMP technique is its potential for the amplification of target DNA under the isothermal condition. The ability of the LAMP method to synthesize a large amount of target DNA is the other advantageous property [35]. Reverse transcription loop-mediated isothermal amplification (RT-LAMP) has been employed to identify infectious diseases [36].

LAMP is a cost-effective alternative to standard PCR since it does not require expensive thermocycler instruments that operate alternating temperatures for the amplification. The method can synthesize up to 109 copies of the target gene in less than an hour. Compared to PCR, a finely tuned LAMP test could be faster and more sensitive to rapid [30]. Another advantage over classical PCR is the direct analysis from swabs without RNA isolation [37].

Additionally, it can be performed at a wide pH range and temperature, and it does not need professional technicians [38]. Moreover, reagents utilized in RT-LAMP are low-cost and stable at 25 °C. Thus, RT-LAMP is a rapid diagnostic analysis to evaluate unknown agents of respiratory illnesses such as COVID-19. Lamb et al. (2020) performed the RT-LAMP technique to detect SARS-CoV-2 under 30 min and developed a diagnostic strategy to mitigate the spreading of COVID-19 [39]. One of the LAMP technique limitations is the intricate primer design, which could impede target site selection [40].

### 5.2 Protein-based detection testing

**Figure 3:**
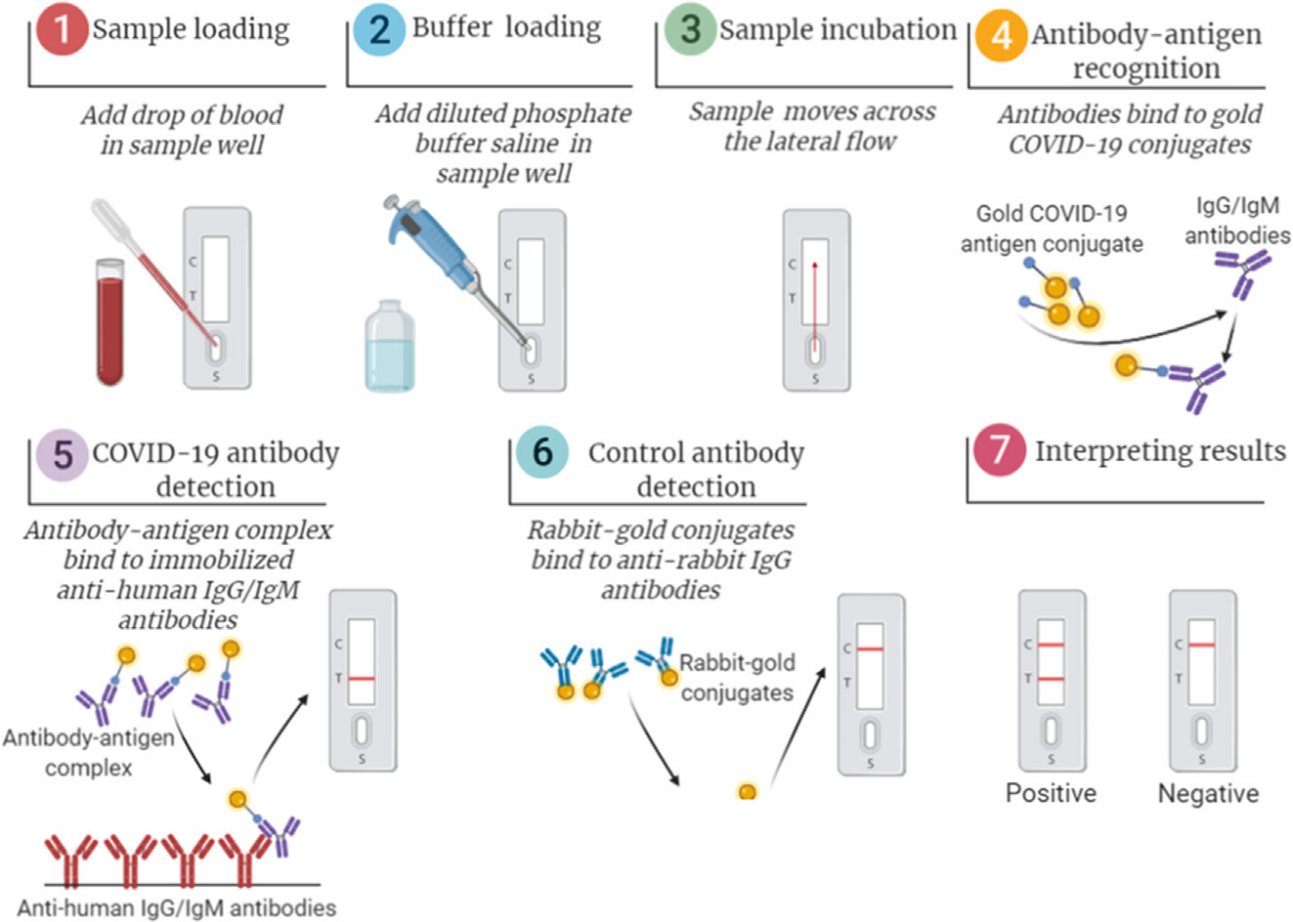
Schematic serological covid-19 detection steps.

The S, N, M, and E proteins are expressed during viral infection, which is very effective amongst the coronavirus structural proteins at prompting antibody responses. The detection of a viral protein antigen, or antibody, which formed as an immune response to the coronavirus infection, is used in COVID-19 diagnosis [41]. However, these methods have some difficulties due to the viral load changes during the infection progress. For example, the first week after the start of the infection shows great salivary viral loads, which progressively decreases in the next weeks [42]. However, the tests based on antibodies are specifically useful in coronavirus diagnosis, there is a concern that can be attributed to the cross-reactivity of SARS-CoV-2 with other antibodies formed in response to separate coronaviruses [43]. As of today, serological tests to detect COVID-19 infections are progressing. Enzyme-linked immunosorbent assay (ELISA) is used to detect immunoglobulin G and M from COVID-19 patient's serum [44]. In the ELISA method, the SARS-CoV-2 Rp3 nucleocapsid protein is used. Recombinant proteins are adsorbed onto plates, and the rest will be thrown away. Then, the patient's serum will be diluted. Following this, antihuman immunoglobulin G is functionalized using horseradish peroxidase and added to the previous solution to capture the target. In the next step, the substrate, 3,3,5,5-tetramethylbenzidine, will be added to the plate, which reacts with peroxidase leading to a detectable color change. In the case of the existence of anti-SARS-CoV-2 immunoglobulin G, a positive signal is formed. A similar process can also be conducted using immunoglobulin M. Recently, researchers at KTH Royal Institute of Technology in Stockholm, Sweden, developed a novel and safe serological test capable of large-scale determining the infection via only a drop of blood analysis (Figure 3) [45]. The technique is based on the detection of antibodies existing in proteins of the individual's blood. Antibody testing can have a mostly complementary role to RT-PCR tests in the diagnosis of COVID-19, at approximately 10 days or more after the onset of symptoms [46,47].

Moreover, rapid antigen detection tests have also been developed to detect active infection. However, in comparison with RT-PCR, rapid antigen detection tests lack sensitivity and owing to the increased risk of false-negative results, they are considered as an adjunct to RT-PCR tests [48].

### 5.3 PoC testing

PoC is a technology that does not require the transportation of samples to laboratories. PoC technique is lateral flow antigen detection which is used to diagnose COVID-19 [49]. In this technique, a membrane is coated in a strip-shape in two parts; the first part is the gold nanoparticle-antibody (GNA) conjugates, which attaches the antibodies in the second part. The proteins of the patient's sample will be pulled gently across the strip through the capillary movement. Once passing the first part, the antigens attach to the GNA and continue together through the membrane. When they reach the second part, they are made stable by capturing antibodies, and subsequently, their color will change to blue or red. It should be noted that a single gold nanoparticle is red, while the accumulation of its nanoparticles is blue, and it is related to the plasmon band.

**Figure 4:**
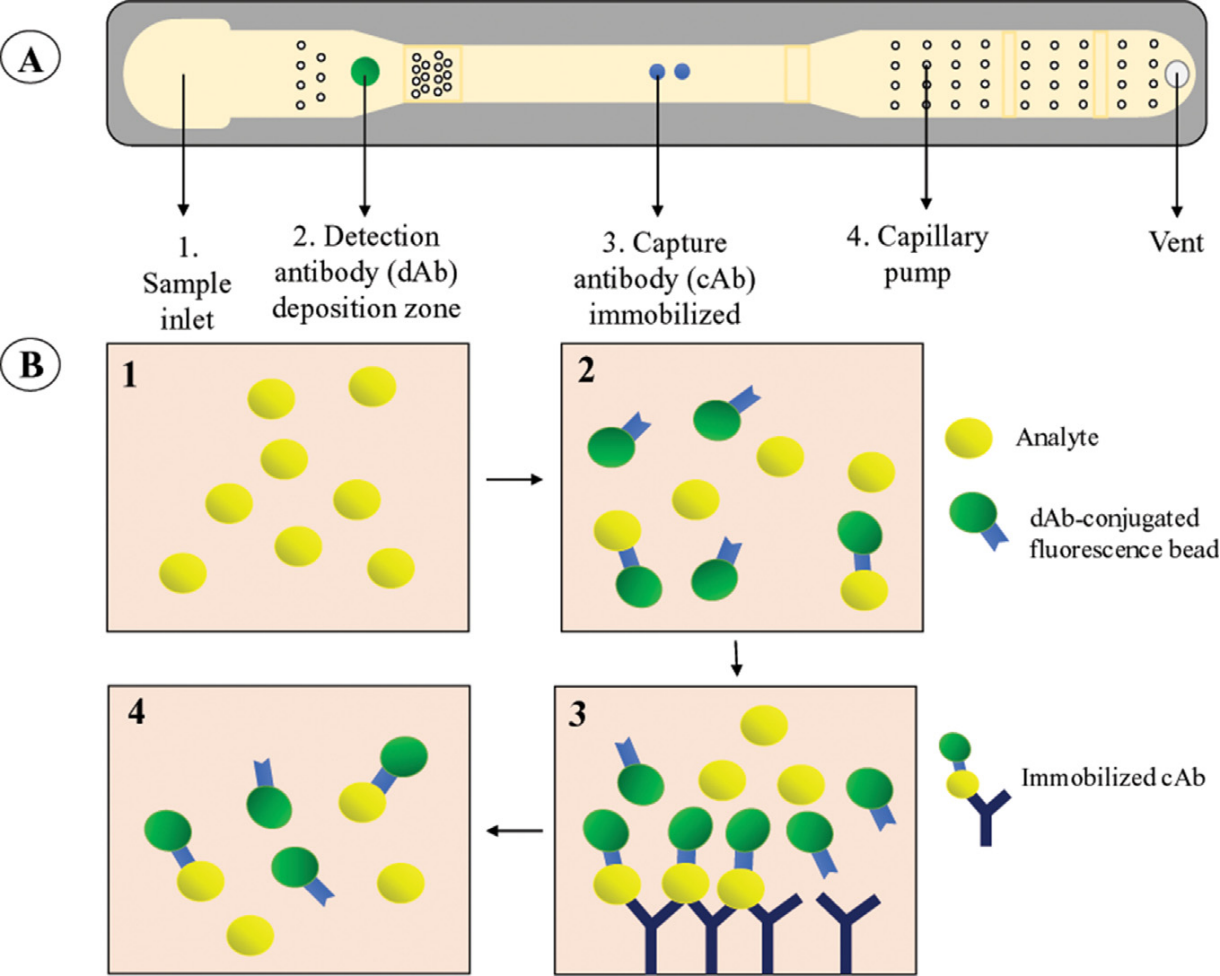
(A) A microfluidic device structure and (B) Simplified interactions along with the flow of sample in a microfluidic device.

The aforementioned technique indicates 100% specificity for both immunoglobulin G and M [12]. In addition, nucleic acid testing can be combined with the lateral flow assay [49,50]. However, these tests are limited due to their low sensitivity compared to RT-PCR. The various signal amplifying methods have been applied to the assay readout to eliminate its signal problem [51]. Exploiting microfluidic devices is another component of PoC. The microfluidic devices contain reaction chambers, a chip, and micro-channels [12]. Figure 4 schematically shows a microfluidic device structure. Utilizing different forces such as vacuum, capillary, etc., enables the chip to combine and separate liquid samples. The materials used in the manufacturing of the chips are mostly glass, paper, or polydimethyl sulfoxide. The crucial benefits of using microfluidics stem from portability, less detection time, and small-sized samples [52]. This technology was used earlier to detect HIV [53], which was completely successful with 100% sensitivity and 87% specificity and now has the potential to be utilized for COVID-19 diagnosis.

Table 1 summarizes all the detection tests with their detailed characteristics to provide better comparisons among them.

**Table 1:** Characteristics of COVID-19 detection methods.

	MOLECULAR TEST (RT-PCR test, LAMP test)	ANTIGEN TEST (Rapid Antigen test)	ANTIBODY TEST (ELISA)
Biomarker	Nucleic Acid	Protein	Protein
Sample type	Nasal or throat swab (most common), Nasopharyngeal, Saliva (least common)	Nasal or Nasopharyngeal swab	Fingerstick or blood draw
Result preparation	Same day or up to a week	Can be speedy (15–30 min)	Same-day or 1–3 days
Accuracy and precision	Highly accurate and mostly does not need to be repeated.	Mostly positive results are highly accurate, but false positives can occur Negative results may need to be proved with a molecular test.	Sometimes a second antibody test is necessary for accurate results.
Diagnostic situation	Active coronavirus infection	Active coronavirus infection	Past coronavirus infection
Disadvantage	Cannot detect past infection	Antigen tests are more likely to miss an active infection compared to molecular tests	Diagnose COVID at the time of the test or show do not have COVID.

## 6 The laboratory diagnosis concerns and solutions

### 6.1 Pre-analytical concerns

SARS-CoV-2 infected patients indicated considerable viral loads in their respiratory tract during the first days of symptoms [54]. For early infection diagnosis, the normal advice is to utilize a nasopharyngeal (NP) swab due to better patient toleration and higher safety for the operator [55]. Moreover, NP swabs have high inherent control in entering the proper location in the nasal cavity. It was reported that NP swabs showed a higher detection rate (63%) compared to oropharyngeal (OP) swabs. Although the collection of either NP or OP may be a useful option for normal conditions, the clinics should consider global supply chains. To get an appropriate NP swab sampling, it should deeply enter the nasal cavity. The wince of the patients is considered as a sign of successful target hitting. Although in the case of obtaining an OP swab, the gag reflex is observed, albeit the gag response varies with people. While spinning the swabs three times, it is recommended to be held in place for 10 s. The lack of personal protective equipment (PPE) can be compensated with a self-collected saliva sample used for the upper respiratory tract sample. Practically, the swab supply seems to be insufficient; hence, self-collected saliva is a great alternative. In the case of a lack of swab supply, an alternative can be non-flocked swabs approved by the Food and Drug Administration (FDA) [17]. The collected swab samples should be placed in a refrigerated condition and quickly transported to laboratories [56]. It is reported that different swabs sometimes miss early infection, which requires either repeating the test or collecting samples of the lower respiratory tract. Repeating the test can be useful, especially when Computed Tomography (CT) scans are ambiguous, and/or viral disease is diagnosed, and/or a patient has a potential exposure history [17]. It is also a challenging decision to make regarding negative test results and the quarantine fate of the suspected individual. In the case of late detection, which led to severe conditions for patients with the worst viral load, sputum or bronchoalveolar lavage are recommended for the sampling of the lower respiratory tract [57].

According to recent research, the best SARS-CoV-2 RNA rate was attributed to bronchoalveolar lavage [58]. It should be noted that in that research, NP swabs were not compared to each other. The highly infected cases of acute respiratory symptoms may need intubation or isolation in a negative pressure place. Ideally, the best time to collect lower respiratory sputum samples is while conducting the intubation process. Otherwise, the samples are collected for both sputum or bronchoalveolar lavage after intubation [59]. The COVID-19 patients showed high viral load in fecal material and delayed shedding of the respiratory tract [60,61]. In some cases, SARS coronavirus was observed in enterocytes. As a result, a rectal swab can be a great alternative option to replace direct respiratory sampling [62].

### 6.2 Analytical concerns

Assays have been used to rapidly detect antigens and/or antibodies related to SARS-CoV-2 [17]. These assays are classified as a technique of PoC testing named lateral flow assays. The advantages of using assays stem from their short detection time as well as cost-efficiency [63]. However, there is a concern for their low sensitivity as they were utilized for influenza earlier [64]. Several antigen assays and monoclonal antibodies are being improved to be used as rapid detection for SARS-CoV-2. Considering different viral loads in infected patients, it is likely that antigen detection does not operate as accurately as expected. Serological measurements demonstrated a great potential for the detection of SARS-CoV-2 [44]. Such measurements indirectly determine the infection and have been successfully used for the detection of other coronaviruses previously [65,66]. Lateral flow assays can effectively measure both immunoglobulin M and G and evaluate the infection extent, basic reproduction number, and overall mortality. However, immunoglobulin M results are not particular and take a long time (several months) to improve for specific responses. Therefore, diagnosis based on serology measurements is not efficient enough to manage the COVID-19 pandemic condition but can be useful for late infected cases such as clinic staff. Moreover, cell culture is not a good option for diagnostic aims [17]. The most recommended molecular diagnostic technique for COVID-19 is rRT-PCR assays [67,68]. Although deep sequencing molecular techniques are needed for the future evolution of SARS-CoV-2 detection and random-amplification deep sequencing techniques are critical in early infection detection, they are currently insufficient in effectiveness for SARS-CoV-2 detection. Other molecular techniques, including LAMP, are being improved worldwide, and some great achievements have occurred so far [69].

WHO's recommendation is E gene assay screening and then confirming with RNA-dependent RNA polymerase gene. It was reported that targeting RNA-dependent RNA polymerase was more successful compared to targeting S and N genes for SARS-CoV-2 detection [70]. In order to restrict cross-reactions and genetic drift, at least two different molecular targets are recommended. Ideally, the design should contain at least one conserved and one specific region to alleviate the potential genetic drift effect that occurs within newly evolved strains of SARS-CoV-2 [17].

### 6.3 Post-analytical concerns

The cycle threshold value for PCR is the cycle number, while fluorescence detection is feasible in higher background signals. A test is positive when the cycle threshold value is lower than 40. When the cycle threshold value is less than 40 for just one of the targets (N1 or N2), it is considered indecisive and requires repeating the test [71]. It should be noted that the results of rRT-PCR should not be considered to decide on either the infection extent or the therapeutic response [72]. Monitoring the infected cases with SARS-CoV-2 is important prior to deciding their isolation or discharge, as they may spread the virus [73]. Thus, it is recommended for up to 30 days of self-quarantine. Both NP and/or OP swabs are not efficient enough to be used for tests of infectivity or cure. Hence, based on retrospective experiences for SARS in 2002, two consecutive negative rRT-PCR tests from rectal swabs are a great option for the test of cure [74]. Considering coronaviruses, there are four different types of proteins in their structure, which are used for SARS-CoV-2 detection through serological techniques. Such techniques mostly detect S protein antibodies, including S1 and S2 [65]. In addition, N protein, with a helical nucleocapsid structure, is critical for the diagnostic procedure of detecting SARS-CoV-2 as it affects RNA packaging and viral pathogenesis. The antibodies against N protein are detected normally in COVID-19 infected cases [75]. Considering Rapid lateral flow assays, although seroconversion was reported for 50% of the infected cases, a quick decrease in viral load was not observed [54]. Overall, serological techniques are effective in the evaluation of the immune condition of asymptomatic infected cases. However, it appears that they may not be feasible for screening or diagnosing early infections [44].

## 7 The role of new technologies in fighting COVID-19

New SARS-CoV-2 detection technologies are developing rapidly worldwide to aid authorities in saving time, energy, and life [76]. The Internet of Things **(**IoT) is one of the new technologies, which refers to connections among things and physical objects equipped with sensors and software to gather information and sending them with the lowest human mediation during the process [77]. IoT technology has been widely exploited in the medical healthcare applications such as therapeutic information administration, telemedicine and remote clinical services, and modern health management [78,79]. In the case of SARS-CoV-2 detection, less human mediation is correlated to a lower risk of individuals being infected. Hence, Mohammed et al. (2020b) devised a system using IoT technology to identify suspected cases of COVID-19. This system contains a smart helmet with mounted thermal and optical imaging systems with minimum human interactions [11]. The smart helmet collects the required information, such as the body temperature of the suspected cases, instead of using one person to do so. The modular system based on IoT communication links announces at a higher temperature by sending a notification. The global positioning system (GPS) module determines the coordinates of the location, and the officer will receive the information of the presumptive COVID-19 patients. Another useful technology in fighting COVID-19 is using smartphones. Interrupting the pandemic of COVID-19 requires extreme and exact tracing, comprehensive patient monitoring, and efficient tools to facilitate communication [80]. Smartphones could help with the aforementioned due to their internet connectivity, computational performance, and ability to analyze electronic and epidemiological reports [81].

Owing to COVID-19's high spread rate, some patients with mild fever and cough symptoms avoid visiting a doctor since the hospitals are frequented by the infected individual having intensive symptoms. In such a scenario, smartphones can efficiently connect the patients with clinicians and lower the risk of being infected in a public place by remote monitoring. Moreover, smartphones facilitate consultation sessions to cope with the hard situation caused by isolation and the fear of being infected [82]. Various mobile apps can be used to report symptoms to the experts and monitor the travel history of people to track the cases effectively. These apps can send notifications and new hygiene tips and protocols during a disease outbreak. Therefore, such devices are useful to address the PoC testing requirements in a wide range. People can report their symptoms to their doctors, and subsequently, the clinicians will consider the symptoms and may ask several questions about them to diagnose initially and decide on further treatments remotely. This process can eliminate unnecessary cases to endanger themselves by entering a risky location.

## 8 Conclusion and future prospects

Accurate and rapid detection systems are important in the early diagnosis of Zoonotic diseases that spread between animals and people, such as SARS and SARS-COV-2, and provide the opportunity for preparedness and mitigation strategies, especially in the case of epidemic and pandemic. At present, the focus of the researchers is on detection methodology suitable for viral RNA. This review aimed to summarized molecular genetic assays, serological assays, and immunological assays for the detection of COVID-19 infection.

For genetic detection methodologies, PCR in general, and RT-PCR in particular, are suitable for RNA viruses. In the selection of the most suitable technology, sensitivity, specificity, and robustness of the detection platform are of prime importance. In the case of SARS-CoV-2, there are extensive mutational changes in S and M proteins, whereas conserved E and N proteins make them potential candidates for the identification of coronavirus. Regarding SARS-CoV-2, we suggest using the multiplex RT-PCR technique based on forward and reverse primer sets targeting several virulent factors. Mitigating the challenges of using multiple primers in the same reaction could be accomplished by avoiding inter-primer homology. Researchers are encouraged to couple genetic detection systems in tandem with serological technologies as a part of a real-time validation strategy.

The sensitive and specific rapid assay is a major focus of assay development in order to speed up the response time for treatment and minimize the waiting time involved with testing. There is an immediate need for accurate and rapid detection of SARS-CoV-2 as a world pandemic disease.

We also propose a built-in combination of genetic and serological platforms based on nanoparticle-coated antibodies and purification of captured viral particles before it serves as a primal matter for the system's genetic detection phase. This strategy reduces the incidences of false-positive and false-negative results. Serological technologies, such as ELISA, inherently possess high speed and ease of use. However, the development and availability of pure and specific monoclonal antibodies is the key. Although LAMP is a viable option for a nucleic acid-based detection system with acceptable sensitivity for detecting DNA or RNA on a small scale and is relatively fast in providing results within 60 min, it is limited by its ability to run process-specific reactions requiring high temperature. In the absence of any laboratory diagnostic testing and emergency cases, CT scans have been used. CT scanner is not expected to detect viral or bacterial particles, but it can detect the signs and symptoms of the invading viruses on the internal organs of an infected person. Therefore, it has very limited application in these cases.

Protein-based detection procedures such as ELISA are designed based on antigen-antibody affinity and reactivity. The system is easy to use while being very rapid, which provides results in minutes. The disadvantage of rapid ELISA platforms is cross-reactivity with nonspecific antibodies and changes in viral load during infection. However, ELISA technology is beneficial for PoC testing. Regardless of the performance of the platform, the reliability of diagnostic results starts with the correct sampling device and sampling technique. NP swab is recommended more than OP swab for early detection due to lower false-positive rate, being tolerated better by presumptively infected individuals, and higher safety. Combining both swabs increases the probability of detection but requires a double number of swabs. Self-collected saliva is recommended as the alternative in case of scarcity of the swabs. Rectal swabs are used in identifying patients with late infection or can be utilized as a test of infectivity/cure, which is of great importance but is currently unacknowledged.

In general, regardless of all challenges and questions about the 2020 pandemic, there is meaningful progress in developing accurate diagnostic assays worldwide. As coronavirus continues to affect human lives in a detrimental manner, the development of rapid and accurate virus detection methods is paramount. In this regard, rRT-PCR, and multiplex RT-PCR detection techniques hold promise.

## Declarations

### Author contribution statement

All authors listed have significantly contributed to the development and the writing of this article.

### Funding statement

This research did not receive any specific grant from funding agencies in the public, commercial, or not-for-profit sectors.

### Data availability statement

No data was used for the research described in the article.

### Declaration of interest statement

The authors declare no conflict of interest.

### Additional information

No additional information is available for this paper.
